# Health care utilization and HIV clinical outcomes among newly enrolled patients following Affordable Care Act implementation in a California integrated health system: a longitudinal study

**DOI:** 10.1186/s12913-020-05856-5

**Published:** 2020-11-11

**Authors:** Derek D. Satre, Sujaya Parthasarathy, Michael J. Silverberg, Michael Horberg, Kelly C. Young-Wolff, Emily C. Williams, Paul Volberding, Cynthia I. Campbell

**Affiliations:** 1 Department of Psychiatry and Behavioral Sciences, Weill Institute for Neurosciences, University of California, 401 Parnassus Avenue, San Francisco, CA 94143 USA; 2 Division of Research, Kaiser Permanente Northern California, 2000 Broadway, 3rd Floor, Oakland, CA 94612 USA; 3 Kaiser Permanente Mid-Atlantic States, Mid-Atlantic Permanente Research Institute, Rockville, MD USA; 4 Health Services Research & Development (HSR&D), Center of Innovation for Veteran-Centered Value-Driven Care, Veteran Affairs (VA) Puget Sound Health Care System, Seattle, WA USA; 5 Department of Health Services, University of Washington, Seattle, WA USA; 6 AIDS Research Institute, University of California San Francisco, San Francisco, CA 94158 USA

**Keywords:** Health care reform, Deductibles, Affordable Care Act, AIDS Drug Assistance Program, Insurance, Mental health, HIV viral suppression, Race/ethnicity

## Abstract

**Background:**

The Affordable Care Act (ACA) has increased insurance coverage for people with HIV (PWH) in the United States. To inform health policy, it is useful to investigate how enrollment through ACA Exchanges, deductible levels, and demographic factors are associated with health care utilization and HIV clinical outcomes among individuals newly enrolled in insurance coverage following implementation of the ACA.

**Methods:**

Among PWH newly enrolled in an integrated health care system (Kaiser Permanente Northern California) in 2014 (*N* = 880), we examined use of health care and modeled associations between enrollment mechanisms (enrolled in a Qualified Health Plan through the California Exchange vs. other sources), deductibles (none, $1–$999 and > = $1000), receipt of benefits from the California AIDS Drug Assistance Program (ADAP), demographic factors, and three-year patterns of health service utilization (primary care, psychiatry, substance treatment, emergency, inpatient) and HIV outcomes (CD4 counts; viral suppression at HIV RNA < 75 copies/mL).

**Results:**

Health care use was greatest immediately after enrollment and decreased over 3 years. Those with high deductibles were less likely to use primary care (OR = 0.64, 95% CI = 0.49–0.84, *p* < 0.01) or psychiatry OR = 0.59, 95% CI = 0.37, 0.94, *p* = 0.03) than those with no deductible. Enrollment via the Exchange was associated with fewer psychiatry visits (rate ratio [RR] = 0.40, 95% CI = 0.18–0.86; *p* = 0.02), but ADAP was associated with more psychiatry visits (RR = 2.22, 95% CI = 1.24–4.71; *p* = 0.01). Those with high deductibles were less likely to have viral suppression (OR = 0.65, 95% CI = 0.42–1.00; *p* = 0.05), but ADAP enrollment was associated with viral suppression (OR = 2.20, 95% CI = 1.32–3.66, *p* < 0.01). Black (OR = 0.35, 95% CI = 0.21–0.58, *p* < 0.01) and Hispanic (OR = 0.50, 95% CI = 0.29–0.85, *p* = 0.01) PWH were less likely to be virally suppressed.

**Conclusions:**

In this sample of PWH newly enrolled in an integrated health care system in California, findings suggest that enrollment via the Exchange and higher deductibles were negatively associated with some aspects of service utilization, high deductibles were associated with worse HIV outcomes, but support from ADAP appeared to help patients achieve viral suppression. Race/ethnic disparities remain important to address even among those with access to insurance coverage.

## Background

Major components of the Affordable Care Act (ACA) [1] were introduced in 2014 to increase access to health insurance coverage in the U.S., particularly for vulnerable populations including people with HIV (PWH). It was expected that mandates of the ACA (e.g., establishment of state insurance exchanges, and inclusion of psychiatric and substance use treatment as essential benefits) implemented in 2014 would increase health care utilization and quality, leading to improved behavioral health and HIV clinical outcomes [2–4].

Early evaluations suggest that some of these expectations have been met. A large multi-state study by the HIV Research Network found a decrease in Ryan White / uncompensated care in Medicaid expansion states post-ACA and a small increase in private coverage in non-expansion states [5]. Among PWH enrolled in the Kaiser Permanente Northern California (KPNC) health care system after ACA implementation, more new enrollees with HIV had a primary care visit within 6 months of enrollment in 2014 compared with a cohort that enrolled in 2012, and rates of HIV viral control improved [6]. Research in Virginia has found improvement in viral suppression following increased access to coverage through the ACA [7, 8]. Similarly, a study based in Nebraska found that insurance enrollment was independently associated with improved health outcomes including viral suppression [9].

However, little is known about the degree to which newly enrolled PWH maintain access to services within healthcare systems and what their HIV clinical outcomes are over time. In particular, increased patient cost sharing via higher deductibles and other changes associated with the ACA such as enrollment via new state insurance exchanges, have the potential to influence care. For example, our previous evaluation of PWH found that new enrollees post-ACA (2014) had more substance use disorders and were more likely to be enrolled in high-deductible plans compared to pre-ACA enrollees (2012) [6], indicating that newly enrolled PWH post-ACA may have greater behavioral healthcare needs as well as financial obstacles to accessing services.

It remains unknown to what extent enrollment through state insurance exchanges and higher deductibles influence outcomes. Exchanges provide access to tax credits, a range of coverage levels, HIV-specific guidance on planning care continuity, and information about medication benefits relevant to PWH that might not be easily accessible through other sources of coverage, e.g., through employers [10, 11], and thus could have a potentially positive impact on subsequent utilization and outcomes. Deductibles may pose a barrier to care, as studies in other populations have shown [12–14].

The AIDS Drug Assistance Program (ADAP) continues to play a role in maintaining access to services, providing medications, and premium and out-of-pocket financial assistance to low-income PWH [15, 16]. A prior study by McManus and colleagues [8] found that Virginia ADAP client enrollment in qualified health plans increased in 2015 compared with the prior year and varied based on demographic and health care delivery factors. Given the potential positive impact of ADAP on access to care even in an insured patient population, we included ADAP in our models along with enrollment via the Exchange and level of deductible.

The current study investigated health care utilization and HIV control in a large health system, KPNC. This system has an integrated model that is becoming increasingly common [17, 18], and follows the standards for health services required of plans offered on the California Exchange [19]. We anticipated that utilization would be greatest immediately after enrollment, reflecting potential pent-up demand for care. We examined factors associated with utilization as conceptualized by the Andersen model [20, 21]. The model proposes that utilization is determined by predisposing (e.g., race/ethnicity and other demographic factors), need (e.g., HIV diagnoses) and enabling factors (e.g., benefit plan, enrollment through the Exchange) [22, 23]. Controlling for demographic factors and ADAP participation, we expected that utilization would be higher and that HIV outcomes would be better among PWH with lower deductibles, and that enrollment via the Exchange also might have a positive effect on these outcomes.

## Methods

### Setting

KPNC is a large, integrated health care system with over 4 million members. Members receive coverage through employers, government programs (e.g., Medicaid and Medicare) and individual plans. KPNC offers comprehensive HIV care. Treatment is provided by HIV specialists integrated into primary care, with support provided by HIV specialty nurses, case managers, and clinical pharmacists. Patients enrolled in ADAP can fill prescriptions directly through KPNC pharmacies. Psychiatry and substance use treatment are available to members [24], as well as emergency department (ED) and inpatient care.

### Study sample and data source

Participants were identified from the KPNC HIV registry, an up-to-date list of HIV-positive patients that includes clinical HIV data and demographic characteristics. The registry is populated through active monitoring of electronic inpatient, outpatient, laboratory, and pharmacy databases for sentinel indicators of HIV infection. HIV-positivity is confirmed through medical record review. The registry has > 24,000 historical PWH, with 8389 active patients during 2014.

Patients included in the present analysis were adults newly enrolled in KPNC between 1/1/2014–12/31/2014 (with no KPNC coverage recorded in the prior 6 months) and were confirmed HIV-positive within 6 months post-enrollment (*N* = 880). Linkage to other databases through medical record identifiers permits analysis of service utilization and HIV outcomes. Study procedures were approved by the University of California San Francisco (UCSF) and KPNC Institutional Review Boards and included waivers of informed consent.

### Measures

We obtained patient characteristics and services use from the electronic health record (EHR), and HIV clinical outcomes data from the HIV registry. Demographic variables included sex, age, and race/ethnicity. Coverage mechanism included enrollment via the California Exchange vs. other mechanisms (e.g., employer-based large group purchasers or individual plans not purchased on the Exchange). KPNC pharmacy databases include codes that indicate whether the fill was subsidized by ADAP; enrollment was assessed from HIV prescription fills in 2014. Deductible limits were classified into 3 levels (none, $1–$999 and > = $1000), as in prior studies within KPNC [25, 26]. Since these may change over time, for analyses we used the values that were applicable at enrollment and every 6 months thereafter.

Utilization measures were aggregated in six-month intervals beginning from the post-intake date for up to 36 months (through 12/31/2017) yielding a maximum of six repeated measures. Total outpatient visit count as well as type (primary care, psychiatry, specialty substance use treatment, ED, inpatient) were summarized for the six time periods.

For HIV clinical outcomes, we created an indicator variable for each six-month period for HIV RNA suppression, defined as HIV RNA levels < 75 copies/mL [27]; on the few occasions when an individual had more than one lab result per time-period, we used the most recent observation. We used continuous measures of CD4 counts for each six-month period, using mean CD4 count in case of multiple measures per six-month period.

### Analyses

Over 36 months post-enrollment, we examined trends in service utilization including visits to primary care (which includes HIV services), psychiatry, substance use treatment, inpatient hospitalization and ED use; and HIV clinical outcomes using bivariate statistics. To account for correlation between repeated measures, we used generalized estimating equations methodology [28]. Using multivariable logistic regression, we examined whether deductible level, enrollment via the Exchange, ADAP and demographic characteristics were associated with use of health care and HIV outcomes in the 3 years post-enrollment. We also examined whether the outcomes differed by gender by including a gender *x* time interaction variable.

We modeled psychiatry and specialty substance use clinic visit counts using the Poisson distribution since we had count data (number of days) with over dispersion of zeroes (no visits) and decreasing probability of having multiple visits. We used the software’s built-in capability to correct for over-dispersion (the dispersion parameter is estimated by the ratio of the deviance to its degrees of freedom). The parameter estimates are not affected but the estimated covariance matrix is inflated by this factor; this is the conventional approach in Poisson regression. We included member months as an offset term to account for varying exposure length (i.e., varying length of membership due to attrition). We used the negative binomial distribution for examining primary care visits based on preliminary analyses of the distribution of visits in these categories. The exponent of the coefficient represents the rate ratio of the utilization measures over time relative to the first six-month post-enrollment period.

The sample had too few Medicaid enrollees to independently examine the effects of Medicaid on study outcomes. However, we conducted a sensitivity analysis with participants who enrolled in the health system via Medicaid removed, to determine if this changed the results of service utilization analyses. All analyses were conducted using SAS v9.4. Significance level was set at 0.05 for all analyses.

## Results

### Sample characteristics

The sample included 880 PWH enrolled in the health care system in 2014 (Table 1). Participants were primarily male (92.6%) and ethnically diverse ethnically diverse (16.6% Blacks, 16.9% Hispanics and 8% Asians), with a mean age of 43.4 years (standard deviation = 10.9 years). Due to small numbers, we combined the American Indian/Alaskan Native and the Native Hawaiian/Pacific Islander groups into “Other” race category in multivariate analyses. Regarding type of health coverage and enrollment, 71.9% had no deductible and 15.9% were enrolled via the California Exchange; 26.6% had ADAP. The proportion of enrollees who obtained coverage via the Exchange and distribution of deductible level was stable over time.

**Table 1:** Demographic characteristics and insurance coverage of newly enrolled health plan members with HIV

	***N*** = 880 n (%)
**Demographic characteristics**	
Gender	
Male	815 (92.6)
Female	65 (7.4)
Age at enrollment	
< 26	45 (5.1)
26 to 35	192 (21.8)
36 to 45	233 (26.5)
46 to 55	288 (32.7)
56 to 64	122 (13.9)
Race/Ethnicity	
White	455 (51.7)
Black	146 (16.6)
Asian	70 (8.0)
American Indian/Alaskan Native	3 (0.3)
Native Hawaiian/Pacific Islander	9 (1.0)
Hispanic	149 (16.9)
Unknown	48 (5.5)
**Deductible level**	
None	541 (71.9)
$1 to $999 (Low Deductible)	77 (10.2)
> =$1000 (High Deductible)	134 (17.8)
**Enrollment mechanism**	
Exchange	140 (15.9)
Other mechanism (e.g., employer, Medicaid)	740 (85.1)
**ADAP Benefit**	213 (24.2)

The sample included PWH newly enrolled in Kaiser Permanente Northern California between 1/1/2014 and 12/31/2014. Payer type included commercial (92.8%), Medicaid (6.6%), and other (0.5%). ADAP = AIDS Drug Assistance Program enrollment in 2014

### Longitudinal trends in services use and HIV clinical outcomes

Nearly all of the sample (97.3%) had at least one primary care visit during the 36-month study period (not shown). Bivariate frequencies showed that the percentage of PWH with a primary care visit declined significantly (*p* < 0.01) from 91.8% in the first 6 months of enrollment to 74.0% by the last six-month period (Table 2a) as a percentage of those who were members during the respective six-month periods. About one-fifth (21.9%) of the sample had at least one psychiatry visit ranging from 9.2% in the first period to 6.6% by year three (trend not significant). During the 36-month study period, 6.3% of individuals had a visit to a substance use clinic, and rates of use were stable over time. Over one-tenth (12.4%) of the sample had an inpatient stay during the 3-year study period. The percentage of people with an inpatient stay ranged from 3.8% in the first 6 months to 2.0% by the last six-month window although no significant decreasing trend was identified. A substantial percentage of PWH (41.1%) had at least one ED visit in 36 months; the rates ranged from 14.9% in the first 6 months to 12.1% in 18–24 months, and to 14.6% by year three. The trend was not statistically significant.

**Table 2:** Unadjusted trends in health services utilization and HIV clinical outcomes over 36 months among newly enrolled members with HIV

	***N*** **= 880**	***N*** **= 824**	***N*** **= 757**	***N*** **= 719**	***N*** **= 677**	***N*** **= 645**
**(a) Services Use**	**0–6 months**	**6–12 months**	**12–18 months**	**18–24 months**	**24–30 months**	**30–36 months**
**(% with Any Use)**						
Primary Care**	91.8%	78.4%	77.0%	74.0%	74.8%	74.0%
Psychiatry	9.2%	8.8%	8.7%	7.8%	6.5%	6.6%
Substance Use Treatment	3.1%	2.8%	2.5%	2.1%	2.5%	2.9%
Inpatient	3.8%	3.8%	4.2%	2.8%	3.3%	2.0%
Emergency	14.9%	13.0%	13.9%	12.1%	12.7%	14.6%
**(b) HIV Clinical Parameters**	**0–6 months**	**6–12 months**	**12–18 months**	**18–24 months**	**24–30 months**	**30–36 months**
Mean CD4 Count**	633.8	639.7	653.2	677.8	682.3	713.9
(Std. Dev.)	(287.6)	(307.5)	(314.3)	(303.2)	(320.4)	(330.1)
HIV RNA level < 75 copies/mL(%)**	84.4%	91.0%	91.1%	93.7%	93.3%	93.1%

Percentages are based on enrolled sample within each 6-month time period. ** *p* < .01. Significance levels for services use and HIV RNA level are based on the chi-squared statistic. Significance levels for mean CD4 count are based on the F-statistic

HIV clinical outcomes improved over time as measured by the increase in the proportion of individuals with HIV RNA control < 75 mL and improvement in CD4 counts (Table 2b). Most of the sample (95.8%) had at least one measure of CD4 count during the 3 years; 95.6% had at least one HIV RNA laboratory measure during this time. Among those who had at least one CD4 measure, mean CD4 count increased significantly (F-test *p* < 0.01) from 633.8 to 713.9 over 3 years. Among those who had an HIV RNA measure, at least 80% had HIV RNA levels < 75 copies/mL in every six-month period; there was an increase over 3 years, from 84.4% in the first 6-month period after enrollment to 93.1% by 3 years (*p* < 0.01).

### Multivariate analyses of service utilization

Logistic regression models including demographic factors, enrollment through the Exchange, deductible level and ADAP did not show any longitudinal trends in dichotomous measures of services use (except for primary care) which suggested that the likelihood of hospitalization, ED use, psychiatry and substance use treatment services was stable over time (not shown). Those with high deductibles were less likely to use primary care (OR = 0.64, 95% CI = 0.49–0.84, *p* < 0.01) or psychiatric services (OR = 0.59, 95% CI = 0.37, 0.94, *p* = 0.03) compared to those with no deductibles. Exchange enrollees were less likely to use psychiatric services (OR = 0.49, 95% CI: 0.28, 0.87, *p* = 0.01), (not shown).

We also examined the number of visits to primary care, psychiatry and substance use departments. There was a decreasing trend in frequency over 36 months (Table 3). The most consistent and significant decreases were in primary care, which declined by 44% (1–0.56) in the 6 to 12 months post-enrollment period relative to the first 6 months after enrollment; this trend continued and by 3 years, participants had 55% fewer visits relative to 0–6 months post-enrollment. Those with high deductibles had 21% fewer primary care visits (RR = .79, 95% CI = 0.71–0.88, *p* < 0.01).

**Table 3:** Multivariate analyses of number of primary care, psychiatry and substance use disorder treatment visits over 36 months among newly enrolled members with HIV

Primary Care Visits	RR (95% CI)	***P***
Time (Reference = 0–6 months)		
6–12 months	0.56 (0.51, 0.61)	<.01
12–18 months	0.51 (0.47, 0.56)	<.01
18–24 months	0.45 (0.41, 0.49)	<.01
24–30 months	0.45 (0.40, 0.49)	<.01
30–36 months	0.45 (0.41, 0.51)	<.01
Gender (Reference = Male)	1.07 (0.86, 1.33)	0.52
Women, 6–12 months	1.61 (1.26, 2.06)	<.01
Women, 12–18 months	1.10 (0.78, 1.54)	0.58
Women, 18–24 months	1.69 (1.15, 2.49)	0.01
Women, 24–30 months	1.91 (1.37, 2.67)	<.01
Women, 30–36 months	1.54 (1.03, 2.30)	0.03
Race/Ethnicity (Reference = White)		
Asian	1.06 (0.83, 1.36)	0.62
Black	1.11 (0.98, 1.26)	0.11
Hispanic	1.09 (0.95, 1.25)	0.22
Other	1.12 (0.77, 1.64)	0.55
Age (at enrollment)	1.01 (1.00, 1.01)	<.01
Deductible level (Reference = none)^a^		
Low deductible	1.03 (0.90, 1.18)	0.67
High deductible	0.79 (0.71, 0.88)	<.01
Exchange (Reference = not through exchange)^b^	1.00 (0.90, 1.11)	0.99
AIDS Drug Assistance Program (Reference = none)	1.10 (0.98, 1.23)	0.10
**Psychiatry Visits**	**RR (95% CI)**	***P***
Time (Reference = 0–6 months)		
6–12 months	1.07 (0.64, 1.78)	0.80
12–18 months	0.64 (0.41, 1.00)	0.05
18–24 months	0.86 (0.44, 1.70)	0.67
24–30 months	0.89 (0.50, 1.60)	0.69
30–36 months	0.65 (0.40, 1.05)	0.08
Gender (Reference = Male)	1.42 (0.55, 3.64)	0.46
Women, 6–12 months	1.97 (0.86, 4.51)	0.11
Women, 12–18 months	2.86 (0.72, 11.43)	0.14
Women, 18–24 months	0.69 (0.25, 1.91)	0.48
Women, 24–30 months	0.32 (0.08, 1.33)	0.12
Women, 30–36 months	0.40 (0.09, 1.79)	0.23
Race/Ethnicity (Reference = White)		
Asian	0.28 (0.10, 0.79)	0.02
Black	1.52 (0.58, 3.98)	0.39
Hispanic	0.73 (0.39, 1.37)	0.33
Other	1.80 (0.60, 5.42)	0.30
Age (at enrollment)	1.03 (1.00, 1.05)	0.04
Deductible level (Reference = none)^a^		
Low deductible	0.96 (0.50, 1.84)	0.89
High deductible	0.52 (0.27, 1.01)	0.05
Exchange (Reference = not through exchange)^b^	0.40 (0.18, 0.86)	0.02
AIDS Drug Assistance Program (Reference = none)	2.41 (1.24, 4.71)	0.01
**Substance Use Treatment Visits**	**RR (95% CI)**	***P***
Time (Reference = 0–6 months)		
6–12 months	0.86 (0.39, 1.89)	0.72
12–18 months	0.89 (0.36, 2.17)	0.79
18–24 months	0.43 (0.17, 1.04)	0.06
24–30 months	0.26 (0.11, 0.65)	<.01
30–36 months	0.19 (0.07, 0.53)	<.01
Gender (Reference = Male)	2.49 (0.55, 11.31)	0.24
Race/Ethnicity (Reference = White)		
Asian	0.48 (0.06, 3.96)	0.49
Black	0.94 (0.25, 3.54)	0.93
Hispanic	0.96 (0.28, 3.30)	0.95
Other	0 (0.00, 0.03)	<.01
Age (at enrollment)	0.98 (0.96, 1.01)	0.25
Deductible level (Reference = none)^a^		
Low deductible	2.31 (0.81, 6.58)	0.12
High deductible	1.32 (0.23, 7.55)	0.75
Exchange (Reference = not through exchange)^b^	1.33 (0.41, 4.33)	0.63
AIDS Drug Assistance Program (Reference = none)	0.48 (0.11, 2.03)	0.32

*RR* Rate ratio, *CI* Confidence interval

^a^Low deductible = $1 to $999; High deductible > = $1000 (comparison = no deductible)

^b^California Insurance Exchange vs. other mechanisms

Psychiatry visits increased in the first year and decreased gradually thereafter; by 12–18 months, individuals were using 36% fewer psychiatry visits (*p* = 0.05) relative to the period immediately after enrollment and by 3 years, the rates were down by 35% (*p* = 0.08). Individuals with high deductibles (RR = 0.52, 95% CI = 0.27–1.01, p = 0.05) were likely to have fewer psychiatry visits compared to those with no deductibles; individuals enrolled through the Exchange (RR = 0.40, 95% CI = 0.18–0.86, *p* = 0.02) had fewer visits compared to those enrolled through other mechanisms. Enrollment in ADAP was associated with greater use of psychiatry (RR = 2.41, 95% CI = 1.24–4.71, *p* = 0.01).

Visits to substance use treatment also declined, but the decreases were larger after 18 months, and by 3 years PWH had 81% fewer substance use treatment visits. Benefit-related factors (enrollment through the Exchange, deductible levels and ADAP) were not significantly associated with visits (Table 3).

#### Multivariate analyses of HIV clinical outcomes

Mean CD4 counts showed a steady increase over time, with coefficients (relative to 0–6 months) increasing from 28.97 in the 6–12 months post-enrollment to 93.68 by 30–36 months (Table 4). All individuals were increasingly likely to have viral control (HIV RNA < 75 copies/mL) over time. Approximately twice as many individuals (OR = 2.06, 1.48–2.89, *p* < .01) were likely to have HIV RNA < 75 copies/mL in the 6–12 months post-enrollment period compared to the first 6 months after enrollment; they were three times as likely (OR = 3.30, 95% CI = 2.01–5.42, *p* < .01) to have HIV RNA < 75 copies/mL by 30–36 months post-enrollment. Those with high deductibles were less likely to have viral suppression (RR = 0.65, 95% CI = 0.42–1.00; *p* = 0.05) compared with those with no deductible, but enrollees in ADAP were more likely to have viral suppression compared to others (OR = 2.20, 95% CI = 1.32–3.66) *p* < 0.01). Black (OR = 0.35, 95% CI = 0.21–0.58, *p* < 0.01) and Hispanic (OR = 0.50, 95% CI = 0.29–0.85, *p* = 0.01) PWH were less likely to be virally suppressed.

**Table 4:** Multivariate analyses of CD4 counts and viral control over 36 months among newly enrolled KPNC members with HIV

CD4 Count	Coefficient (95% CI)	P
Time (Reference = 0–6 months)		
6–12 months	28.97 (1.48, 2.89)	<.01
12–18 months	46.8 (1.49, 3.29)	<.01
18–24 months	63.21 (1.73, 3.91)	<.01
24–30 months	69.54 (1.93, 4.63)	<.01
30–36 months	93.68 (2.01, 5.42)	<.01
Gender (Reference = Male)	14.3 (0.27, 1.02)	0.76
Race/Ethnicity (Reference = White)		
Asian	−1.22 (0.45, 2.06)	<.01
Black	−62.62 (0.21, 0.58)	0.05
Hispanic	−79.72 (0.29, 0.85)	<.01
Other	48.12 (0.08, 1.56)	0.62
Age (at enrollment)	−2.13 (1.00, 1.04)	0.03
Deductible level (Reference = none)^a^		
Low deductible	−14.62 (0.36, 1.11)	0.33
High deductible	13.36 (0.42, 1.00)	0.36
Exchange (Reference = not through exchange)^b^	15.28 (0.54, 1.42)	0.27
AIDS Drug Assistance Program (Reference = none)	27.37 (1.32, 3.66)	0.26
**HIV RNA level < 75 Copies/mL**	**OR (95% CI)**	**P**
Time (Reference = 0–6 months)		
6–12 months	2.06 (1.48, 2.89)	<.01
12–18 months	2.22 (1.49, 3.29)	<.01
18–24 months	2.6 (1.73, 3.91)	<.01
24–30 months	2.99 (1.93, 4.63)	<.01
30–36 months	3.3 (2.01, 5.42)	<.01
Gender (Reference = Male)	0.52 (0.27, 1.02)	0.06
Race/Ethnicity (Reference = White)		
Asian	0.97 (0.45, 2.06)	0.93
Black	0.35 (0.21, 0.58)	<.01
Hispanic	0.5 (0.29, 0.85)	0.01
Other	0.36 (0.08, 1.56)	0.17
Age (at enrollment)	1.02 (1.00, 1.04)	0.04
Deductible level (Reference = none)^a^		
Low deductible	0.63 (0.36, 1.11)	0.11
High deductible	0.65 (0.42, 1.00)	0.05
Exchange (Reference = not through exchange)^b^	0.88 (0.54, 1.42)	0.59
AIDS Drug Assistance Program (Reference = none)	2.2 (1.32, 3.66)	0.01

*KPNC* Kaiser Permanente Northern California, *OR* Odds Ratio, *CI* Confidence interval

^a^Low deductible = $1 to 999; high deductible = $1000+ (comparison = no deductible)

^b^California Insurance Exchange vs. other mechanisms

#### Longitudinal trends by gender

**Fig. 1:**
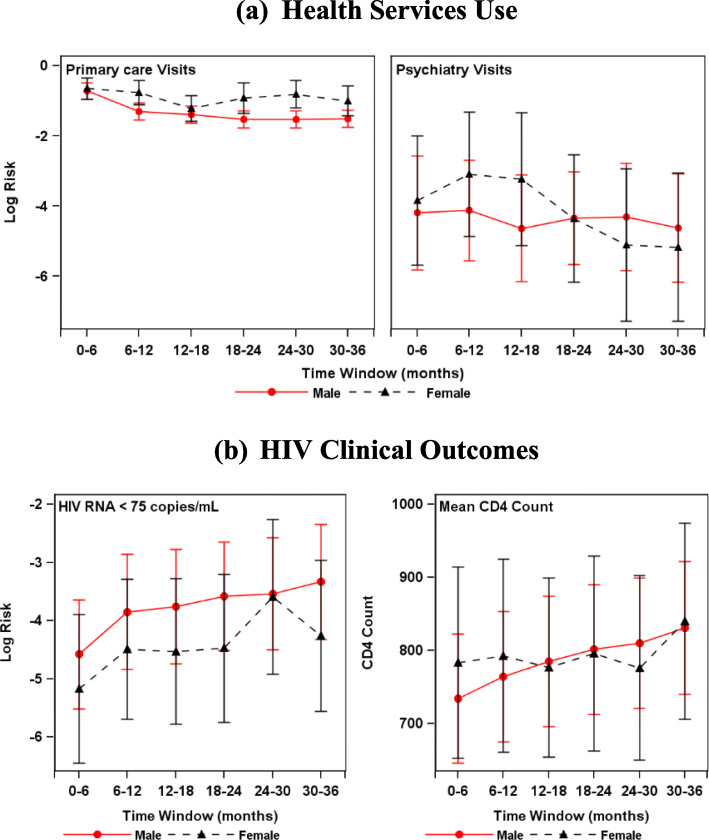
Plot of Health Services Utilization and Clinical Outcomes - Gender x Time

Women were higher utilizers of primary care services than men during the 3 years of the study but psychiatry services use did not differ by gender (Fig. 1). Due to small cell sizes (e.g., there were no substance use services visits by women in some time windows) we did not examine gender *x* time interactions in substance use treatment. Clinical HIV outcome trajectories did not differ by gender.

#### Sensitivity analysis with Medicaid enrollees removed

The utilization models were repeated after excluding those with Medicaid, with a few minor changes noted: In analysis of predictors of number of primary care visits, the effect of ADAP enrollment became significant (RR = 1.16 (95% CI = 1.03,1.30, *p* = .01), rather than non-significant (*p* = .11, Table 3). In analysis of predictors of number of psychiatry visits, high deductible became non-significant (RR = 0.55, 95%CI = 0.26, 1.14, *p* = .10) rather than borderline significant (*p* = .05, Table 3). All other coefficients in all the other utilization models were substantively the same (i.e., significance or insignificance of the coefficients remained the same).

## Discussion

This study examined patterns of health service utilization post-ACA among PWH newly enrolled in insurance coverage in a large integrated health care system in California. Overall, our study findings indicated that use of health care was highest immediately after enrollment; that coverage through the Exchange and deductible level had some relationship to service utilization, although associations varied by service; and high deductibles were associated with worse viral control. ADAP benefits were associated with access to psychiatry and better HIV viral control.

We found that most of the study sample (91.8%) had a primary care visit within 6 months of enrollment. Since primary care in this health system included HIV treatment, this is an encouraging indicator that KPNC staffing and services were adequate overall for PWH soon after implementation of the substantial health policy changes and enrollment increases associated with the ACA. Consistent with prior studies examining utilization [29, 30], we found that primary care utilization was highest immediately following enrollment and then decreased. Women used more primary care services over time than men and had comparable trajectories of clinical improvement, in potential contrast to a recent study in the Veterans Affairs Health System that found worse HIV care engagement among women [31]. Apart from deductibles, a factor contributing to having fewer primary care visits over time may be improvement in antiretroviral effectiveness [32, 33], which has led to a decrease in the recommended frequency of laboratory testing among individuals with long-term stable viral suppression.

It is important for patients and providers to understand how coverage policies affect services for PWH. Our finding that higher deductibles were associated with worse viral control is consistent with prior findings on how deductibles have the potential to affect access to care [34]. ADAP financial support could play a role in offsetting the effects of higher deductibles on access to care [35], but an association of deductibles with utilization and viral control was still present in our results. Prior work in the KPNC health care system found that HIV care coordinators make an active effort to “onboard” newly enrolled PWH, including ADAP enrollment [6], which could have had a positive impact on management of costs associated with deductibles as well as linkage to psychiatry. It is not known if other health systems made such efforts. One prior study found that increased out-of-pocket spending on antiretroviral therapy associated with Medicare Part D enrollment did not have an impact on HIV viral suppression [35]. These results suggest that PWH who face higher deductible costs, at least in some health systems, have been able to manage these obligations without compromising viral suppression.

We examined the relationship of enrollment through the Exchange to outcomes because enrollment mechanisms are likely to remain a major focus of health care policy in the U.S. Efforts were made on the part of some California health systems [6] and the Exchange itself [10] to educate new members (and potential members) on tiers of coverage and health care initiation processes. Although it is not known if other states made similar efforts, the Exchange in California represented a novel mechanism of coverage and entailed potential challenges both in determining how to choose coverage and sign up (pre-enrollment) and in understanding benefits and services (post-enrollment) [4]. Our findings that enrollment through the Exchange made no difference in accessing primary care or in HIV outcomes for newly enrolled PWH suggests that regardless of mechanism, new enrollees were able to access core services.

One recent study in California found that those with employer-based insurance had greater access to providers than those with individual private insurance plans or Medicaid [36]. Consistent with our psychiatry utilization findings, this study found worse access to care among those with private coverage purchased on exchanges compared to private coverage purchased individually [36]. Another study found that patients often felt overwhelmed by the array of choices offered on the exchanges and were confused by terminology and websites [37]. The reason for the association of enrollment through the Exchange with psychiatry utilization in our sample could also be due to higher cost-sharing in Exchange plans (apart from deductibles) or to unexamined group differences such as financial constraints or fewer mental health problems among Exchange vs. non-exchange enrollees.

It is worth noting that we found worse HIV outcomes for Black and Hispanic PWH, despite similar utilization of primary care, mental health, and substance use treatment. We conducted post-hoc analyses to explore whether this pattern might be attributable to people with poorer viral control utilizing more services. We found a significant negative correlation between viral control and number of viral load tests (*ρ* = 10%; *p* < .01), and between viral control and primary care visits (*ρ* = 10%; *p* < .01); in addition, PWH with poorer viral control had 1.04 (Std. error = 0.19) more visits over 3 years than those with adequate viral control. Race/ethnic disparities are a longstanding concern in the HIV treatment field. It was hoped that disparities would be at least partially mitigated post-ACA [4, 38], although recent data indicate that Black and Hispanic PWH continue to have worse HIV care outcomes across multiple health care settings [39–41]. It is possible that factors associated with race/ethnicity and worse HIV outcomes not measured in our study such as health literacy [42] and variability in use of electronic provider communication tools [43, 44] could have contributed to these differences. The race/ethnic disparities in HIV clinical outcomes observed in our sample, in which overall insurance coverage was not a barrier to care, highlight the importance of addressing this ongoing challenge to health equity.

### Study strengths and limitations

The study was conducted in a large integrated health system with access to data on type of insurance coverage, enrollment mechanisms, ADAP, use of health services and routine laboratory measures; and measured outcomes over 3 years following ACA implementation. However, limitations should be noted. The California Exchange continues to modify its coverage options [45, 46], and the effects of enrollment via the Exchange on outcomes of interest are likely to shift over time. Data on insurance coverage and viral control prior to KPNC enrollment were not available. Loss to follow up is also a limitation: although retention was high, HIV outcomes were based on PWH who remained in care and could be high compared to those who did not complete routine laboratory testing or left the health plan during study follow-up.

Although Medicaid expansion has benefitted PWH [47, 48], too few Medicaid beneficiaries were identified to examine separately. Yet repeating our analyses with Medicaid beneficiaries excluded, as a sensitivity analysis, resulted in few changes to the results. There is variability in the ways that patients can use ADAP in California (i.e., support for medication purchases as well as help in purchasing insurance and covering co-pays) [49], which we were not able to examine. However, we included ADAP status using prescription records as an indicator of whether study participants had received financial assistance through this program.

Insurance coverage policies relevant to HIV care may vary by state [50], limiting generalizability. The sample was drawn from a single institution in California and participants had relatively high levels of viral control relative to PWH in other settings such as Ryan White clinics [41, 51]. However, economic barriers to service utilization for PWH with access to care are of concern in many health systems, including systems in other states and countries, and our study contributes to this important area of inquiry. In addition, conducting the study in single large integrated health care allowed us to examine our research questions without needing to control for variability across health care insurance and treatment providers.

## Conclusions

This study examined factors associated with use of health care and HIV control among PWH newly enrolled in health insurance coverage following ACA implementation in a California health system. Outpatient utilization was highest immediately after enrollment and subsequently decreased. Higher deductibles and enrollment through the Exchange were negatively associated with psychiatric services. HIV clinical outcomes improved over time and were associated with ADAP enrollment; but having a high deductible was associated with lower odds of viral suppression. Race/ethnic disparities in HIV clinical outcomes remain critical to address among PWH, even in integrated health plans.

## Data Availability

The datasets generated and/or analyzed during the current study, including the HIV Registry [6], are not publicly available (closed). The study team received administrative permission from Kaiser Permanente to use these datasets. The data that support the findings of this study are available from Kaiser Permanente but restrictions apply to the availability of these data, which were used under license for the current study, and so are not publicly available. Data are however available from the authors upon reasonable request and with permission of Kaiser Permanente. Active partnerships between external researchers and the parent study team facilitates more productive collaborations that will effectively leverage the investment to develop the data as compared with generation of public use datasets. Upon request, de-identified data will be made available as study manuscripts are published. However, there remains the possibility of deductive disclosure of participants with unusual characteristics and disclosure of Kaiser Permanente proprietary information. Thus, researchers who seek access to individual level data will be required to sign a data sharing agreement. After the end of the project funding period, the cost of computer programmers preparing de-identified datasets suitable for sharing will be borne by the party requesting the data.

## Abbreviations

ACA: Affordable Care Act
ADAP: AIDS Drug Assistance Program
ED: Emergency department
EHR: Electronic health record
KPNC: Kaiser Permanente Northern California
PWH: People with HIV
SAS: Statistical Analysis Software
UCSF: University of California, San Francisco
